# Bacterial vaginosis in female facility workers in north-western Tanzania: prevalence and risk factors

**DOI:** 10.1136/sti.2008.035543

**Published:** 2009-05-26

**Authors:** K Baisley, J Changalucha, H A Weiss, K Mugeye, D Everett, I Hambleton, P Hay, D Ross, C Tanton, T Chirwa, R Hayes, D Watson-Jones

**Affiliations:** 1 London School of Hygiene & Tropical Medicine, London, UK; 2 National Institute for Medical Research (NIMR), Mwanza, Tanzania; 3 African Medical and Research Foundation (AMREF), Mwanza, Tanzania; 4 St George’s Hospital, London, UK

## Abstract

**Objectives:**

To determine prevalence of, and risk factors for, bacterial vaginosis (BV) among herpes simplex virus (HSV) 2 seropositive Tanzanian women at enrolment into a randomised, placebo-controlled trial of HSV suppressive treatment.

**Methods:**

1305 HSV-2 seropositive women aged 16–35 years working in bars, guesthouses and similar facilities were interviewed, examined and tested for HIV, syphilis, *Neisseria gonorrhoeae*, *Chlamydia trachomatis*, BV, candidiasis and trichomoniasis. Factors associated with BV were analysed using logistic regression to estimate odds ratios and 95% confidence intervals.

**Results:**

BV prevalence was 62.9%; prevalence of Nugent score 9–10 was 16.1%. Independent risk factors for BV were work facility type, fewer dependents, increasing alcohol consumption, sex in the last week (adjusted OR 2.03; 95% CI 1.57 to 2.62), using cloths or cotton wool for menstrual hygiene, HIV (adjusted OR 1.41; 95% CI 1.09 to 1.83) and *Trichomonas vaginalis* infection. There was no association between BV and the frequency or method of vaginal cleansing. However, BV was less prevalent among women who reported inserting substances to dry the vagina for sex (adjusted OR 0.44; 95% CI 0.25 to 0.75).

**Conclusion:**

BV was extremely prevalent among our study population of HSV-2 positive female facility workers in North-western Tanzania. Although recent sex was associated with increased BV prevalence, vaginal drying was associated with lower BV prevalence. Further studies of the effects of specific practices on vaginal flora are warranted.

Bacterial vaginosis (BV) is one of the most common vaginal infections worldwide. It is characterised by vaginal flora changes, including a decrease in lactobacilli, particularly those that produce hydrogen peroxide, and an increase in anaerobic and facultative anaerobic bacteria. BV has been associated with a variety of adverse health outcomes, including preterm delivery,^1^ intrauterine infection,^1^ pelvic inflammatory disease (PID)^2^ and other gynaecological complications.^3^

BV may also increase susceptibility to HIV and other sexually transmitted infections (STIs),^4^^–^6 and in prospective studies BV has been associated with the acquisition of HIV and herpes simplex virus (HSV) type 2.^7^^–^11 BV may also facilitate HIV and HSV-2 transmission by increasing the frequency of genital shedding of the virus.^12^

The aetiology of BV remains poorly understood, although sexual activity and vaginal hygiene practices have been associated with BV.^4^ ^13^^–^16 Reproductive hormones are also thought to play a role in the regulation of vaginal flora.^17^ ^18^ In sub-Saharan Africa, the prevalence of BV is very high, ranging from 30–51% in community based studies.^6^ ^10^ A high prevalence has also been reported among African–American women with estimates of up to 50% in population-based surveys.^19^ The reasons for this are unclear. Prevention of BV, and any impact that this might have on reducing the risk of HIV acquisition, will depend in part on identification of risk factors for BV that are susceptible to interventions.

We report on the factors associated with BV prevalence at baseline in a cohort of women at high risk of STI working in bars, guesthouses and similar facilities in Tanzania. These women were enrolled into a trial of the effect of HSV-2 suppressive treatment on HIV incidence, and on genital HIV and HSV-2 viral shedding in women who are HIV positive.

## MATERIALS AND METHODS

### Study population

A randomised, double-blind, placebo-controlled trial of acyclovir 400 mg twice daily suppressive treatment was conducted in women HSV-2 seropositive in the Lake Victoria region of Tanzania. In total, 1305 women aged 16–35 years working in bars, guesthouses and similar facilities were enrolled in three phases from January 2004 to May 2006. Trial procedures have been described previously.^20^ Screening, enrolment and 3-monthly follow-up visits were conducted at mobile clinics established in 19 communities in high HIV-transmission areas. Women were followed for 12–30 months depending on phase of enrolment.

At screening, after informed consent, participants were tested for antibodies to HSV-2 and HIV, and interviewed about sociodemographic details, sexual behaviour and intravaginal practices (IVP). Eligible women were given an appointment to attend the mobile clinic 8–12 weeks later; women were asked to avoid vaginal cleansing before attending the visit. To be eligible, women had to be HSV-2 seropositive, have no history of epilepsy, able to give informed consent, not planning to move out of the study site, not breastfeeding and not pregnant or planning pregnancy during follow-up.

At enrolment, participants were tested for pregnancy and were asked about recent sexual behaviour and IVP. Women were examined for symptoms of reproductive tract infections (RTI), and blood and genital samples were collected. Participants were offered syndromic management for any RTI diagnosed at enrolment, risk reduction counselling, family planning and condoms.

All women, including those ineligible to enrol, were offered free HIV voluntary counselling and testing, with onward referral to the nearest centre providing HIV care, including anti-retroviral treatment where appropriate.

### Laboratory methods

Screening sera were tested for HSV-2 using a type-specific IgG ELISA (Kalon Biologicals, Guildford, Surrey, UK). Screening and enrolment sera were tested for HIV by dual ELISA (Murex HIV Ag/Ab Combination ELISA, Murex Biotech, Dartford, UK; Uni-FormII Ag/Ab micro ELISA system, bioMérieux, Basingstoke, UK). Repeatedly discordant samples were tested by HIV-1 p24 Ag EIA (Biorad Genetic Systems, California, USA) and line immunoassay (INNO-LIA HIV I/II, Innogenetics, Gent, Belgium), as described previously.^20^ Enrolment samples were tested for syphilis by the rapid plasma reagin (RPR) test (Omega Diagnostics, Alva, Scotland, UK) and *Treponema pallidum* particle agglutination (TPPA) assay (Fujirebio Inc, Tokyo, Japan), and by fluorescent treponemal antibody assay (Trepo-Spot IF, bioMérieux, Maray-l'Etoile, France) if RPR positive and TPPA negative.

For BV, vaginal smears were heat-fixed and Gram-stained then examined by light microscopy by a single reader and scored using the Nugent criteria.^21^ External quality control of a random sample of 10% of slides was performed by an external reader (PH). *Trichomonas vaginalis* was diagnosed by wet preparation and culture (InPouch TV, BioMed Diagnostics, San Jose, California, USA). Wet preparations were examined by light microscopy for *Candida albicans* spores and hyphae. *Chlamydia trachomatis* and *Neisseria gonorrhoeae* were diagnosed by the AmplicorCT/NG PCR assay (Roche Diagnostics Systems, Branchburg, New Jersey, USA). *N gonorrhoeae* PCR-positive samples were retested in duplicate and classified using a “two-out-of-three” strategy, and an optical density ⩾2 was used as the cut-off for positive rather than the manufacturer’s recommended value of ⩾0.2.

Cervico-vaginal lavage (CVL) from all HIV positive women were tested for HIV-1 RNA and HSV DNA by real-time PCR using the ABI-7300 system (Applied Biosystems, Courtabeouf, France) and manual nucleic acid extraction (Nuclisens miniMAG bioMérieux, Marcy l’Etoile, France). In addition, CVL from a random sample of 449 HIV negative women were tested for HSV using the same method.

### Statistical methods

Risk factors for BV were analysed using odds ratios (OR) and 95% confidence intervals (CI) obtained by logistic regression. Normal and intermediate flora categories (Nugent score 0–6) were combined to form the category “absence of BV”. Unclassifiable slides containing blood, mucus and/or pus were excluded.

Potential determinants of BV infection were considered using a conceptual framework^22^ with four groups: socio-economic factors, sexual behavioural factors, IVP and menstruation, and biological factors. Age, considered an a priori confounder, was included in all multivariate models. An initial model included socio-economic factors adjusted for age. Factors whose association reached statistical significance at p<0.1 were included in a multivariate model and those that remained independently associated with BV (p<0.1) were retained. The association between each determinant in the sexual behaviour group was assessed by adding the single determinant into the multivariate socio-economic model. Sexual behavioural factors whose association reached significance at p<0.1 were included in a multivariate model; those which remained significantly associated with BV (p<0.1) were retained. Associations with factors in the IVP group and in the biological group were assessed in the same way. The final model was reached by excluding factors one at a time until all remaining factors were significant at the p<0.05 level.

## RESULTS

### Study population

Results at screening have been described previously.^20^ In total, 1305 HSV-2 seropositive women were enrolled. Excluding 22 women with unclassifiable vaginal smears, 807 (62.9%) women had BV, 287 (22.4%) had intermediate changes on Nugent score and 189 (14.7%) had normal flora. Among women with BV, 207 (25.7%) had a Nugent score of 9–10.

Overall, 37.1% of women were HIV seropositive, 13.8% had evidence of active syphilis, 5.3% had gonorrhoea, 7.0% had *C trachomatis* and 30.3% had trichomoniasis. HSV genital shedding was detected in 34 (7.6%) HIV negative and 70 (14.5%) HIV positive women. Among HIV seropositive women, 52.6% had detectable HIV shedding.

At least one symptom of a RTI was reported by 40.3% of women. There was no association between any reported clinical symptom and BV prevalence, and 60.7% of women with BV claimed to be asymptomatic. On examination, women with BV were more likely to have a diagnosis of vaginal discharge syndrome (OR 1.24; 95% CI 0.99 to 1.57).

### Socio-economic factors and association with BV

The mean age of participants was 27.6 years (SD 4.9); 54% were divorced, widowed or separated, and 86% had at least one child. About half (51%) were local food handlers and 16% were bar workers. Over half (53%) reported drinking alcohol each week. BV was most prevalent in younger women, with 71.8% of those aged 16–19 years having BV compared with 60.9% of those aged over 25 years. Prevalence was highest among bar workers and guesthouse workers (71.0% and 73.2%, respectively, compared with 59.3% of local food handlers; table 1). A multivariate model of socio-economic factors adjusted for age showed that type of facility and decreasing number of dependents were independently associated with BV.

**Table 1:** Association of socio-economic and behavioural factors with bacterial vaginosis (BV) infection

Factor	BV prevalence n/N (%)	Univariate OR (95% CI)	Adjusted OR* (95% CI)
Socio-economic factors			
Age (years)		p = 0.02	p = 0.36
16–19	51/71 (71.8%)	1	1
20–24	191/285 (67.0%)	0.80 (0.45 to 1.41)	0.94 (0.51 to 1.73)
25–29	259/418 (62.0%)	0.64 (0.37 to 1.11)	0.76 (0.42 to 1.40)
30–35	306/509 (60.1%)	0.59 (0.34 to 1.02)	0.81 (0.44 to 1.49)
Marital status		p = 0.07	p = 0.58
Married/living with partner	188/324 (58.0%)	1	1
Divorced/separated/widow	441/693 (63.6%)	1.27 (0.97 to 1.66)	1.16 (0.87 to 1.55)
Unmarried	178/266 (66.9%)	1.46 (1.04 to 2.05)	1.17 (0.78 to 1.75)
Facility type		p = 0.002	p = 0.07
Local food handler	389/656 (59.3%)	1	1
Restaurant/café/grocery	116/196 (59.2%)	1.00 (0.72,1.38)	0.87 (0.62,1.23)
Guesthouse	101/138 (73.2%)	1.87 (1.25 to 2.82)	1.65 (1.08 to 2.52)
Local brew seller	59/93 (63.4%)	1.19 (0.76 to 1.87)	0.96 (0.59 to 1.56)
Bar	142/200 (71.0%)	1.68 (1.19 to 2.37)	1.25 (0.86 to 1.83)
Education		p = 0.82	p = 0.42
Primary or above	570/909 (62.7%)	1	1
Less than primary	237/374 (63.4%)	1.03 (0.80 to 1.32)	0.89 (0.68 to 1.18)
Literacy		p = 0.09	p = 0.28
Yes	608/986 (61.7%)	1	1
No	199/297 (67.0%)	1.26 (0.96 to 1.66)	1.18 (0.87 to 1.58)
Number of dependents†		p<0.001	p = 0.003
⩾2	466/785 (59.4%)	1	1
1	142/224 (63.4%)	1.19 (0.87 to 1.61)	1.14 (0.82 to 1.59)
None	199/274 (72.6%)	1.82 (1.34 to 2.46)	1.68 (1.20 to 2.34)
Behavioural factors			
Number of drinks per week		p<0.001	p = 0.001
0	347/602 (57.6%)	1	1
1–4	192/299 (64.2%)	1.32 (0.99 to 1.76)	1.33 (0.98 to 1.81)
5–9	90/137 (65.7%)	1.41 (0.95 to 2.07)	1.47 (0.97 to 2.22)
⩾10	178/245 (72.7%)	1.95 (1.41 to 2.70)	1.75 (1.21 to 2.51)
Age at first sex‡		p = 0.001	p = 0.009
⩾18 y	213/368 (57.9%)	1	1
16–17 y	276/448 (61.6%)	1.17 (0.88 to 1.55)	1.11 (0.83 to 1.49)
14–15 y	246/375 (65.6%)	1.39 (1.03 to 1.87)	1.28 (0.94 to 1.75)
<14 y	63/82 (76.8%)	2.41 (1.39 to 4.20)	2.15 (1.21 to 3.81)
No. partners in last 3 months		p<0.001	p = 0.59
0	29/63 (46.0%)	1	1
1	413/690 (59.9%)	1.75 (1.04 to 2.94)	1.16 (0.66 to 2.05)
2	210/323 (65.0%)	2.18 (1.26 to 3.76)	1.28 (0.70 to 2.34)
⩾3	155/207 (74.9%)	3.49 (1.94 to 6.28)	1.47 (0.75 to 2.85)
Condom use‡		p = 0.007	p = 0.59
Never	132/244 (54.1%)	1	1
Rarely/sometimes	359/570 (63.0%)	1.44 (1.07 to 1.96)	1.15 (0.83 to 1.60)
Often	143/209 (68.4%)	1.84 (1.25 to 2.70)	1.34 (0.87 to 2.05)
Always	172/259 (66.4%)	1.68 (1.17 to 2.41)	1.22 (0.82 to 1.83)
Sex in exchange for money or gifts in past 3 months		p<0.001	p = 0.20
No	464/790 (58.7%)	1	1
Yes	343/493 (69.6%)	1.61 (1.27 to 2.04)	1.19 (0.91 to 1.54)
Hormonal contraception		p = 0.009	p = 0.09
No	587/900 (65.2%)	1	1
Yes	220/383 (57.4%)	0.72 (0.56 to 0.92)	0.80 (0.62 to 1.04)
Sex in past 7 days		p<0.001	p<0.001
No	200/393 (50.9%)	1	1
Yes	607/890 (68.2%)	2.07 (1.62 to 2.64)	1.95 (1.52 to 2.50)

*Adjusted for age, facility type, number of dependents, number of drinks per week, age at first sex, hormonal contraception and sex in the past 7 days; †number of adults and children for whom respondent is contributing to their food and shelter; ‡data for age of first sex are missing for 10 women who could not remember. Data for condom use are missing for 1 woman.

### Association of BV with sexual and other behavioural factors

The majority of women (71%) reported fewer than 10 lifetime partners and 54% reported only 1 partner in the past 3 months. Most participants (64%) reported never or rarely using condoms and 38% reported having had sex in exchange for money or gifts in the past 3 months. At enrolment, 30% were using hormonal contraception; among those, 62% used injectable depot-medroxyprogesterone acetate (DMPA).

On univariate analysis, BV was significantly associated with a number of sexual risk factors, including younger sexual debut, increasing partners in the past 3 months, increasing condom use, paid sex in the past 3 months and sex in the past week (table 1). After adjusting for age and socio-economic factors, only younger age at first sex, increasing alcohol consumption and recent sex remained independently associated with BV. There was some evidence of an inverse association between BV and hormonal contraception (adjusted OR 0.80; 95% CI 0.62 to 1.04).

### Association of BV with intravaginal practices

Vaginal cleansing was reported by 65% of women of whom 99% reported hygiene to be the primary reason for cleansing and 90% used soap and water only. The mean cleansing frequency was 2.6 times/day (range 1–7). Using vaginal lubricants, such as petroleum jelly, during sex (wet sex) was reported by 10% of women. Only 5% reported inserting substances such as lemon or caustic soda to reduce lubrication during sex (dry sex). Although asked to avoid IVP before the clinic visit, 68% of women reported cleansing or inserting substances for sex in the previous 24 hours. Mean time since last IVP was 9.3 hours (range 1–24) for cleansing and 10.6 hours (4–24) for other IVP. Most women (78%) reported using cloths for menstrual protection; 4% reported using cotton wool.

In the univariate analysis, BV was not associated with frequency or timing of vaginal cleansing nor the methods used (table 2). In a multivariate model of IVP, adjusted for socio-economic and sexual behavioural factors, BV was significantly lower among women who reported ever inserting substances for dry sex (adjusted OR 0.45; 95% CI 0.26 to 0.78), although there was no association between BV and vaginal drying in the past 24 hours. BV was higher among women using cloths or cotton wool for menstrual hygiene. There was a non-significant trend towards decreased BV prevalence with increased frequency of vaginal cleansing (adjusted p = 0.10), but no evidence of a trend with reported time since last cleansing.

**Table 2:** Association of intravaginal practices (IVP) and biological factors with bacterial vaginosis (BV) infection

Factor	BV prevalence n/N (%)	Univariate OR (95% CI)	Adjusted OR* (95% CI)
Intravaginal practices			
Vaginal cleansing†		p = 0.79; p trend = 0.34	p = 0.36; p trend = 0.10
Does not cleanse	292/451 (64.7%)	1	1
Once daily	47/74 (63.5%)	0.95 (0.57 to 1.58)	0.95 (0.56 to 1.62)
Twice daily	174/282 (61.7%)	0.88 (0.64 to 1.19)	0.78 (0.56 to 1.08)
⩾3 times/day	293/473 (61.9%)	0.89 (0.68 to 1.16)	0.80 (0.60 to 1.07)
Usual method of cleansing†		p = 0.39	p = 0.20
Does not cleanse	292/451 (64.7%)	1	1
Water only	86/142 (60.6%)	0.84 (0.57 to 1.23)	0.78 (0.52 to 1.18)
Soap & water	368/600 (61.3%)	0.86 (0.67 to 1.11)	0.78 (0.60 to 1.02)
Other products	60/87 (69.0%)	1.21 (0.74 to 1.98)	1.11 (0.66 to 1.87)
Insert substances for wet sex†		p = 0.23	p = 0.99
No	722/1158 (62.3%)	1	1
Yes	84/124 (67.7%)	1.27 (0.85 to 1.88)	1.00 (0.66 to 1.52)
Insert substances for dry sex†		p = 0.008	p = 0.004
No	777/1220 (63.7%)	1	1
Yes	29/62 (46.8%)	0.50 (0.30 to 0.84)	0.45 (0.26 to 0.78)
Any IVP in last 24 h‡		p = 0.81; p trend = 0.81	p = 0.89; p trend = 0.99
No	254/413 (61.5%)	1	1
13–24 h ago	149/230 (64.8%)	1.15 (0.82 to 1.61)	1.05 (0.74 to 1.49)
7–12 h ago	164/255 (64.3%)	1.13 (0.82 to 1.56)	1.12 (0.80 to 1.57)
⩽6 h ago	240/385 (62.3%)	1.04 (0.78 to 1.38)	0.98 (0.73 to 1.33)
Menstrual hygiene†		p = 0.05	p = 0.02
Sanitary pads	135/229 (59.0%)	1	1
Cloths/underwear/sponges	623/988 (63.1%)	1.19 (0.89 to 1.59)	1.34 (0.97 to 1.85)
Cotton wool/toilet paper	39/51 (76.5%)	2.26 (1.13 to 4.55)	2.53 (1.23 to 5.24)
Last menstrual period†		p = 0.38	p = 0.40
<7 days	216/350 (61.7%)	1	1
7–13 days	197/296 (66.6%)	1.23 (0.89 to 1.71)	1.23 (0.88 to 1.74)
⩾14 days	382/612 (62.4%)	1.03 (0.79 to 1.35)	1.02 (0.77 to 1.36)
Biological factors			
HIV		p<0.001	p = 0.002
No	483/813 (59.4%)	1	1
Yes	324/470 (68.9%)	1.52 (1.19 to 1.93)	1.49 (1.16 to 1.92)
*Trichomonas vaginalis†*		p<0.001	p = 0.002
No	530/889 (59.6%)	1	1
Yes	274/390 (70.3%)	1.60 (1.24 to 2.06)	1.54 (1.17 to 2.01)
*Neisseria gonorrhoeae*†		p = 0.13	p = 0.60
No	758/1215 (62.4%)	1	1
Yes	48/67 (71.6%)	1.52 (0.88 to 2.62)	1.16 (0.65 to 2.07)
*Chlamydia trachomatis*†		p = 0.37	p = 0.11
No	754/1193 (63.2%)	1	1
Yes	52/89 (58.4%)	0.82 (0.53 to 1.27)	0.69 (0.44 to 1.08)
Candidiasis†		p = 0.61	p = 0.59
No	718/1145 (62.7%)	1	1
Yes	87/134 (64.9%)	1.10 (0.76 to 1.60)	1.12 (0.75 to 1.65)
HIV genital shedding§		p = 0.14	p = 0.20
No	161/223 (72.2%)	1	1
Yes	162/246 (65.9%)	0.74 (0.50 to 1.10)	0.76 (0.50 to 1.15)
HSV-2 genital shedding¶		p = 0.98	p = 0.77
No	513/811 (63.3%)	1	1
Yes	65/103 (63.1%)	0.99 (0.65 to 1.52)	0.93 (0.60 to 1.46)

*Adjusted for age, facility type, number of dependents, number of drinks per week, age at first sex, hormonal contraception and sex in the past 7 days; †data for vaginal cleansing are missing for 3 women. Data for inserting substances for sex are missing for 1 woman. Data for menstrual hygiene are missing for 15 women. Date of last menstrual period could not be remembered by 25 women. Results for *Neisseria gonorrhoeae* and *Chlamydia trachomatis* missing for 1 woman. Results for *Trichomonas vaginalis* and candidiasis missing for 4 women; ‡cleansing vagina or inserting substances for sex; §among 469 women HIV positive; ¶among 914 women (445 HIV negative and 469 HIV positive).

### Association of BV with biological factors

On univariate analysis, BV prevalence was significantly associated with HIV and *T vaginalis* infection but not with any other STI or with HIV or HSV genital shedding (table 2). After adjusting for socio-economic, behavioural and IVP factors, HIV and *T vaginalis* remained significantly associated with BV.

### Final independent risk factors for BV

In the final multivariate model, independent risk factors for BV were facility type, having fewer dependents, increasing alcohol consumption, recent sex, using cloths or cotton wool for menstrual hygiene, and prevalent HIV and *T vaginalis* infection (table 3). BV was inversely associated with ever inserting substances for dry sex. Age was retained in the final model, although it was no longer significantly associated with BV.

**Table 3:** Final multivariate model for factors associated with bacterial vaginosis (BV) infection

	BV prevalence n/N (%)	Adjusted OR* (95% CI)
Age (years)		p = 0.23
16–19	51/71 (71.8%)	1
20–24	191/285 (67.0%)	0.87 (0.48 to 1.60)
25–29	259/418 (62.0%)	0.66 (0.37 to 1.20)
30–35	306/509 (60.1%)	0.73 (0.40 to 1.33)
Facility type		p = 0.03
Local food handler	389/656 (59.3%)	1
Restaurant/café/grocery	116/196 (59.2%)	0.90 (0.64 to 1.28)
Guesthouse	101/138 (73.2%)	1.82 (1.18 to 2.81)
Local brew seller	59/93 (63.4%)	0.92 (0.56 to 1.51)
Bar	142/200 (71.0%)	1.26 (0.85 to 1.86)
Number of dependents		p = 0.001
⩾2	466/785 (59.4%)	1
1	142/224 (63.4%)	1.15 (0.82 to 1.60)
None	199/274 (72.6%)	1.75 (1.25 to 2.44)
Number of drinks per week		p = 0.002
0	347/602 (57.6%)	1
1–4	192/299 (64.2%)	1.36 (1.00 to 1.85)
5–9	90/137 (65.7%)	1.44 (0.94 to 2.19)
⩾10	178/245 (72.7%)	1.76 (1.22 to 2.55)
Sex in past 7 days		p<0.001
No	200/393 (50.9%)	1
Yes	607/890 (68.2%)	2.03 (1.57 to 2.62)
Inserting substances for dry sex†		p = 0.003
No	777/1220 (63.7%)	1
Yes	29/62 (46.8%)	0.44 (0.25,0.75)
Menstrual hygiene†		p = 0.02
Sanitary pads	135/229 (59.0%)	1
Cloths/underwear/sponges	623/988 (63.1%)	1.42 (1.02 to 1.95)
Cotton wool/toilet paper	39/51 (76.5%)	2.52 (1.21 to 5.25)
HIV		p = 0.008
No	483/813 (59.4%)	1
Yes	324/470 (68.9%)	1.41 (1.09 to 1.83)
*Trichomonas vaginalis*†		p<0.001
No	530/889 (59.6%)	1
Yes	274/390 (70.3%)	1.59 (1.22 to 2.09)

*Adjusted for all variables in the table; †data for inserting substances for dry sex are missing for 1 woman. Data for menstrual hygiene are missing for 15 women. Results for *Trichomonas vaginalis* are missing for 4 women.

## DISCUSSION

We report an extremely high prevalence of BV among Tanzanian HSV-2 seropositive women working in bars, guesthouses and similar facilities, with 63% of women having BV. Other studies in similar populations in sub-Saharan Africa have reported BV prevalence between 20 and 50%.^5^ ^6^ ^8^ ^23^ ^24^ The higher BV prevalence observed in our study is likely to reflect the fact that our population was HSV-2 seropositive, since a strong association between BV and HSV-2 has been observed in many studies.^5^ ^7^ ^23^

We found BV prevalence to be independently associated with sex in the past 7 days. Other studies have found BV to be associated with increased sexual activity in both community based settings and among women at high risk of STI.^15^ ^16^ ^23^ However, a prospective study of rural women in The Gambia found no association between sexual behaviour and BV,^25^ and BV has also been detected in sexually inexperienced women.^19^ A recent meta-analysis found the epidemiological data to be consistent with BV having a sexual mode of transmission,^15^ but additional factors are likely to contribute to its aetiology.

Vaginal cleansing was common in our study population as reported in a number of African studies.^6^ ^13^ ^23^ ^26^ ^27^ IVPs are believed to alter the dominant flora of the vagina and, thus, increase susceptibility to BV.^14^ ^27^ ^28^ IVP, particularly douching, has been described as a risk factor for BV in industrialised countries.^14^ ^28^ In sub-Saharan Africa, the association between IVP and BV is inconsistent, perhaps reflecting the heterogeneity of the techniques and substances used. In a prospective study of sex workers in Burkino Faso, vaginal cleansing was not associated with BV.^23^ Among family planning clinic attendees in Zimbabwe and Uganda, vaginal cleansing was not associated with BV although vaginal drying was associated with BV in the univariate analysis.^27^ Interestingly, we found BV prevalence was significantly lower among women who reported inserting drying substances, although that practice was relatively uncommon. Furthermore, we found a suggestion of a trend towards decreasing BV with increasing frequency of vaginal cleansing.

Menstrual hygiene practices have also been suggested as an explanation for the higher prevalence of BV in sub-Saharan Africa. BV prevalence was higher among women in our study who used cloths or cotton wool than in those who used sanitary pads. However, a study in Uganda found no association between BV and using cloths for menstrual hygiene.^26^ Furthermore, a crossover trial in The Gambia of modern pads and traditional cloths found BV to be slightly more frequent when women used sanitary pads.^25^

Although not statistically significant, we found some evidence of a protective effect of hormonal contraception on BV, which is consistent with other studies.^18^ ^27^ The relationship between hormones, contraception and BV is not well-understood. Oestrogen increases vaginal epithelial cell activity, resulting in increased glycogen and a more favourable environment for lactobacilli.^17^ However, the prevalence of BV among post-menopausal women is low despite low oestrogen levels.^17^

We found strong independent associations of BV with HIV and *T vaginalis*. The association between BV and HIV acquisition has been reported in several prospective studies in sub-Saharan Africa.^9^ ^11^ ^27^ In a similar cohort in Tanzania, women with BV at baseline had a twofold higher risk of HIV seroconversion than those without BV.^8^ However, a trial of presumptive STI treatment to reduce HIV acquisition showed a marked decrease in BV without any impact on HIV,^29^ and HIV infection may be associated with increased prevalence and persistence of BV.^24^

There are several limitations to our analysis. This is a cross-sectional analysis and so we cannot determine causality of the observed associations. Residual confounding may have remained when adjusting for self-reported risk factors, especially sexual behaviours, which are difficult to measure accurately. The risk factors for BV that we identified may not be generalisable since the women in our study were at high risk of other RTIs and all were HSV-2 positive. Although women were asked to avoid vaginal cleansing before they attended the clinic many reported cleansing in the past 24 hours, which possibly reduced the accuracy of our diagnosis of BV. However, we found no association between women’s reported time of last cleansing and BV. Strengths of our study include a large sample size and the use of an expert microbiology laboratory with external quality control to diagnose BV.

Given the association of BV with HIV acquisition shown in many studies, and the extremely high prevalence of BV in this and other African populations, a high proportion of HIV infections may be attributable to BV. Further research is warranted on preventable risk factors for BV, including specific vaginal practices that may alter the vaginal flora.

**Key messages**

- Bacterial vaginosis (BV) prevalence was extremely high in this cohort of HSV-2 positive women.
- Consistent with studies in industrialised countries and Africa, we found strong independent associations of BV with HIV, *Trichomonas vaginalis* and recent sex.
- Vaginal cleansing was very common in this cohort, but we found no association between BV and the frequency, timing or methods of cleansing.
- BV prevalence was significantly lower among women who reported inserting vaginal drying substances, although that practice was relatively uncommon.
